# Sleep patterns, problems, and habits in a sample of Egyptian preschoolers

**DOI:** 10.5935/1984-0063.20220016

**Published:** 2022

**Authors:** Maha K. Abou-Khadra, Dalia Ahmed, Samar A. Sadek, Hala H. Mansour

**Affiliations:** 1 Faculty of Medicine, Cairo University, Pediatrics - Cairo - Cairo - Egypt.; 2 Faculty of Medicine, Cairo University, Public Health - Cairo - Cairo - Egypt.

**Keywords:** Child, Preschool, Sleep, Epidemiology

## Abstract

**Objective:**

Sleep problems are common among preschoolers. We conducted this study to investigate sleep problems in a sample of Egyptian preschoolers attending pediatric outpatient clinics and examine the relationship between their sleep problems, patterns, and hygiene.

**Methods:**

The parents of 319 preschoolers, aged 2-5 years, completed the BEARS(which represent the fve major sleep domains, i.e., bedtime problems, excessive daytime sleepiness, awakenings during the night, regularity and duration of sleep, and snoring) questionnaire in Arabic and a short survey on their educational status, significant medical problems and/or their child’s medications , and sleeping habits.

**Results:**

The frequency of bedtime problems, excessive daytime sleepiness, awakenings during the night, regularity of sleep, and snoring were 58.9%, 17.9%, 31%, 60.5%, and 20.4%, respectively. More than a third of the samples had poor sleep hygiene practices, ranging from 41.7% to 70.5%. Multivariate analyses revealed that age and body mass index (BMI) are predictors of bedtime problems.

**Conclusions:**

Our fndings indicate that sleep problems and poor sleep hygiene are common among this sample of preschoolers. This study also suggests an association between age and BMI and sleep disturbances.

## INTRODUCTION

Sleep problems in infants and young children are common and often underdiagnosed.^1^ Many studies have found a high prevalence of sleep problems among preschoolers.^2,3^ A number of studies have demonstrated high prevalence of sleep problems among school children in Arab countries.^4,5^ However, little is known about the prevalence of sleep problems among preschoolers in this region. Mindell et al. conducted a study to describe sleep patterns and problems in a large sample of infants and toddlers (from birth to 3 years) and their mothers in Arabic-speaking families in the Middle East. The study reported that a significant percentage of parents perceived that their child had sleep problems (37%), with a high prevalence of poor sleep in mothers (72%), and both young children and their mothers have delayed sleep schedules.^6^ Several studies reported poor sleep hygiene among children and adolescents.^7,8^ A recent study that examined sleep patterns and habits among children aged 1-14 years revealed that 36.1% of children 3 - 5 years old had televisions in the bedroom, 65.5% of them used video devices before sleeping, and 33.1% of 1-14 years old children did not follow sleep duration recommendations.^9^ The American Academy of Sleep Medicine recommended that the required amount of sleep to promote optimal health for preschoolers is 10 -13 hours per 24 hours (including regular naps).^10^ Another study that examined the association between electronic media use and sleep pattern among preschoolers revealed that electronic media use was associated with later bedtimes, later wake times, increased daytime napping, and less consolidated sleep.^11^ Several studies reported an association between sleep parameters and weight status among preschoolers.^12,13^ A short sleep duration of ≤10 hours was associated with increased BMI z score in a large sample of Australian preschoolers.^12^ Because of the high prevalence of sleep problems among children, the American Academy of Pediatrics recommends screening children who snore to prevent and minimize the morbidity associated with sleep-disordered breathing.^14^ The prevalence of diagnosed sleep disorders in the large pediatric primary care network was 3.7%.^15^ The BEARS (which represent the five major sleep domains, i.e., bedtime problems, excessive daytime sleepiness, awakenings during the night, regularity and duration of sleep, and snoring) is an easy-to-use pediatric sleep screening tool that significantly increases the amount of recorded sleep information, increasing the likelihood of characterizing sleep problems in primary care settings.^16^ The Persian version of the BEARS was used to detect sleep problems among Iranian children aged 2-14 years after asking parents global question on their children’s sleep. Using the BEARS questionnaire, it was revealed that 45.2% (136/301) of the children had one or more sleep disorders despite the parents reporting sleep disorders by only 9.9% in the primary global question.^17^ Another recent study demonstrated that the BEARS was a useful screening tool for sleep problems among children with monosymptomatic enuresis. ^18^ In the present study, we aimed to investigate sleep disturbances in a sample of preschoolers attending outpatient clinics using simple screening tool and examine the sleep habits of this sample and the possible predictors of their sleep problems. We hypothesized that sleep problems are common among Egyptian preschoolers, and we expect a significant association between sleep disturbances and habits.

## MATERIAL AND METHODS

### Study design and participants

Using purposive sampling, this cross-sectional study recruited 319 children aged 2-5 years from the general outpatient clinics at the Pediatric Hospital, Cairo University. We included those who met the selection criteria using the calculated sample size and excluded those who had medical disorder, neurological disorder, and comorbid psychiatric disorder or were taking medications known to affect sleep. The parents of the preschoolers underwent a brief survey on their educational status, significant medical problems and/or medication for children, sleep patterns included bedtimes and wake times on weekdays and weekends and nap duration to calculate the total sleep time, and unhealthy sleep habits (i.e., sleep hygiene behaviors). Sleep hygiene behaviors include behaviors such as taking caffeine within 4 hours before bedtime, using a computer or mobile device, TV watching, exercising or doing heavy physical activity, being exposed to bright light within 1 hour before bedtime, and sleeping with lights on. We used close-ended questions on unhealthy sleep habits that parents responded to. For parents with little or no formal schooling, we read the questions and provided a detailed explanation. BMI was calculated by measuring the weight and height of the child on the day of data collection. The weights of the children aged 2-5 years old who were not wearing shoes were recorded using EKS 8632 VI scale (EKS Asia Ltd., Hong Kong, China). The heights were measured using a tape measure after confirming the highly accurate position. The children were classified as underweight, normal, overweight, and obese based on the World Health Organization BMI percentiles.^19^ Underweight was defined as BMI ˂5th percentile for age and sex, normal was defined as BMI between the 5th and <85^th^ percentile, overweight was defined as BMI between the 85th and <95^th^ percentile, and obesity was defined as BMI ≥95^th^ percentile.^20^

Data were collected from November 2018 to February 2019. This study was approved by the research committee of the Department of Pediatrics, Faculty of Medicine, Cairo University. Ethical approval was obtained from the Institutional Review Board for Human Subject Research at Faculty of Medicine, Cairo University, Egypt. Verbal consent was obtained from the parents of the children who participated in the study.

### Measure of sleep patterns and problems

The Arabic version of the BEARS questionnaire for the age group of 2-5 years was used after translation and back translation by the Centre for Foreign Languages and Professional Translation, Cairo University. The BEARS is a screening tool developed to address the most common sleep issues. It incorporates five major sleep domains: bedtime problems, including difficulty going to bed and falling asleep; excessive daytime sleepiness, including behaviors typically associated with daytime somnolence in children; awakenings during the night; regularity of sleep/wake cycles (bedtime and wake time) and average sleep duration; and snoring. These domains appear to reflect the most common presenting sleep complaints in children. This screening tool prompts clinicians to ask parents an initial screening question on the possible problems in each domain, eliciting a yes or no response. If the answer was „yes“, then the parents were asked to describe the problem.^16^ Sleep patterns include bedtime and wake time during weekdays and weekends and nap duration to calculate the total sleep duration.

### Study variables

The dependent variables were the five sleep domains in the BEARS questionnaire and sleep patterns. The independent variables were gender, age groups, BMI percentiles, unhealthy sleep habits, and parents’ educational status as an indicator of socioeconomic status. Variables with p-values of less than 0.05 were selected for further multivariate analyses to detect the predictors of sleep disturbances.

### Statistical analysis

Data were analyzed using Statistical Package for the Social Sciences (version 21, IBM Corp., Armonk, NY, USA) and summarized using numbers and percentages for qualitative variables. The mean and standard deviation were used for normally distributed quantitative variables, while the median and interquartile range (IQR) were used for non-normally distributed quantitative variables. Comparisons between groups were conducted using the chi-square test for qualitative variables, whereas the nonparametric Kruskal - Wallis test, and Mann -Whitney U test were used for non-normally distributed quantitative variables. Correlation was conducted to test the linear relation between the quantitative variables. Logistic regression analysis was performed to test the significant predictors of binary dependent variables, and linear regression was performed to test the significant predictors of quantitative variables. A p-value < 0.05 was considered significant.

## RESULTS

### Sample characteristics

The characteristics of the study population are shown in Table 1.

**Table 1:** Characteristics of the studied sample.

Characteristics	Mean ± SD	N (%)
Age	3.4 ± 0.9	
**Gender**		
Male		152 (47.6)
Female		167 (52.4)
**BMI**	16.4 ± 2.7	
**BMI percentiles**		
Underweight		35(11.0)
Normal		158(49.5)
Overweight		54(16.9)
Obese		72(22.6)
**Father education**		
Illiterate		53 (16.6)
High school or less		77 (24.1)
University or above		189 (59.3)
**Mother education**		
Illiterate		66 (20.7)
High school or less		69 (21.6)
University or above		184 (57.7)
**Sleep habits**		
Caffeine intake		133 (41.7)
Exercise or heavy physical activities		136 (42.6)
Television watching		225 (70.5)
Computer or mobile use		163 (51.1)
Bright light exposure		190 (59.6)
Sleeping with the light on		33 (10.3)

### Sleep/wake patterns

The sleep patterns of the study population are shown in Table 2. The median and IQR of total sleep duration on weekdays and weekends were the same (11 and 10-12 hours, respectively). The median and IQR of wake time were 8:00 and 7:00-9:30 on weekdays and 9:00 and 7:00-10:00 on weekends, respectively; the median and IQR of bedtime were 22:00 and 21:00-00:00 on weekdays and 23:00 and 22:00-00:00 on weekends, respectively. There were significant negative correlations between wake time on weekdays (r=−0.112, p=0.045), bedtime on weekdays (r=−0.112, p=0.046), total sleep duration on weekends (r=−0.159, p=0.004), and age, while there was no significant correlation between sleep patterns and BMI (p>0.05). Bedtime on weekdays was significantly later (p=0.033) in boys than in girls as the median and IQR were 23:00 and 21:00-00.00 and 22:00 and 21:00-00:00, respectively. There was no significant difference in sleep patterns regarding the paternal level of education, while there was a significant difference in nap duration regarding the maternal level of education (children whose mothers had high school or low level of education: median, 1 hour; IQR, 1-2 hours; children whose mothers had little or no formal schooling: median, 2 hours; IQR, 1-2 hours; children whose mothers had university or above level of education: median, 2 hours; IQR, 1-2 hours ; p=0.042).

**Table 2:** Sleep Pattern of the studied sample.

Sleep Pattern	Median (IQR)
**Wake up time, h: min.**	
Weekdays	8:00 (7:00 - 9:30)
Weekends	9:00 (7:00 - 10:00)
**Bedtime, h: min.**	
Weekdays	22:00 (21:00 - 00:00)
Weekends	23:00 (22:00 - 00:00)
**Nocturnal sleep duration, h**	
Weekdays	10.0 (9.0 - 11.0)
Weekends	10.0 (9.0 - 11.0)
**Nap duration, h**	2.0 (1.0 - 2.0)
**Total Sleep duration, h**	
Weekdays	11.0(10.0 - 12.0)
Weekends	11.0(10.0 - 12.0)

## FREQUENCY OF SLEEP PROBLEMS

The frequencies of the BEARS items are shown in Table 3. The most frequent problem among the studied sample was bedtime problems (58.9%), while the least frequent one was excessive daytime sleepiness (17.9%). Regarding age groups, there were significant differences in bedtime problems and regularity of sleep in 2- year-olds; they have the highest frequency of bedtime problems (76.6%), followed by 3- year- olds (60.7%), while 4- and 5- year-olds had the least frequency (48 %and 52.6%, respectively), with a statistically significant difference (p=0.003). Children aged 4-years had the highest frequency of regularity of sleep (70%), followed by children aged 3- years (60.7%), while children aged 2- and 5- years had the least frequency (48.4 % and 55.3%, respectively), with a statistically significant difference (p=0.044). There was a significant gender difference regarding snoring (p=0.012) as it was reported more frequently in boys (26.3%) than in girls (15%). There was no statistically significant difference in sleep problems among different BMI percentile groups, except for bedtime problems (p=0.017), as it was less frequent among underweight children (34.3%) compared with normal weight (62%), overweight (64.8%) and obese (59.7%) children. Regularity of sleep was significantly higher (p<0.001) among children whose fathers had university or above education (72.5%) compared with children whose fathers had little or no formal education (39.6%) and had high school or less education (45.5%). Similarly, regularity of sleep was significantly higher (p<0.001) among children whose mothers had university or above education (72.3%) compared with children whose mothers had little or no formal education (39.4%) and had high school or less education (49.3%).

**Table 3:** Sleep problems according to BEARS questionnaire among the studied sample.

BEARS questionnaire items	Median (IQR)	N (%)
**Bedtime problems**		188 (58.9)
**Excessive daytime sleepiness**		57 (17.9)
Taking nap		161 (50.5)
**Awakenings during night**		99 (31.0)
**Regularity and duration of sleep**		193 (60.5)
Wake up time	8:00 (7:00 - 9:30)	
Bedtime	22:00 (21:00 - 00:00)	
**Snoring**		65 (20.4)

### Impact of sleep habits on sleep patterns and the frequency of sleep problems

Regarding sleep patterns, caffeine intake had a significant impact on bedtime (caffeine intake: median, 23:00; IQR, 21:00-00:00; no caffeine intake: median, 22:00; IQR, 21:00-23:00;p=0.004) and wake time: (caffeine intake: median, 8:00; IQR, 7:00-10:00; no caffeine intake: median, 8:00 ;IQR, 6:53-9:00; p=0.035 ) on weekdays. Similarly, TV watching had a significant impact on bedtime (TV watching: median, 22:30; IQR, 21:00-00:00; no TV watching: median, 22:00; IQR, 20:53-23:00; p=0.016) and wake time: (TV watching: median, 8:00; IQR, 7:00-10:00; no TV watching: median, 7:30; IQR, 6:53-8:38; p=0.018) on weekdays. Computer or mobile use led to a significantly latter bedtime on weekends (computer or mobile use: median, 23:00 ;IQR, 22:00-01:00; no computer or mobile use: median, 22:30; IQR, 21:00-00:00; p=0.002). Exposure to bright light prior to bedtime led to a significantly later bedtime (exposure to bright light: median, 23:00; IQR, 21:00-00:00; no exposure to bright light: median, 22:00; IQR, 21:00-23:00; p=0.015), shorter nocturnal sleep duration (exposure to bright light: median, 10 hours; IQR, 9-11 hours; no exposure to bright light: median, 10 hours; IQR, 9-11 hours; p=0.027), and shorter total sleep duration(exposure to bright light: median, 10.25 hours; IQR, 9.5-11.5 hours; no exposure to bright light: median, 11 hours; IQR, 10-12; p=0.002) on weekdays. Sleeping with light on led to earlier wake time on weekdays ( sleeping with light on: median, 7:00; IQR, 6:15-8:30; sleeping without light on: median 8:00; IQR, 7:00-9:30; p=0.026) and weekends (sleeping with light on : median, 7:30; IQR, 6:30-10:00; sleeping without light on: median, 9:00; IQR, 8:00-10:30; p=0.014); it also led earlier bedtime on weekends ( sleeping with light on: median, 22:00; IQR, 21:00-00:00; sleeping without light on: median, 23:00; IQR, 22:00-00:00; p=0.030). There was no significant difference in sleep patterns regarding exercise or heavy physical activity prior to bedtime (p>0.05). Regarding sleep problems, there were significant differences in both awakenings during night (p=0.002) and regularity of sleep (p=0.028) between children who take and don’t take caffeine before bedtime, with a significantly higher frequency of awakenings during night with caffeine intake before bedtime (40.6% vs. 24.2%) and a lower frequency of regularity of sleep with caffeine intake before bedtime (53.4% vs. 65.6%). The frequency of regularity of sleep was significantly lower (p=0.02) among children exposed to bright light before bedtime (55.3%) compared with those not exposed (68.2%). TV watching, computer or mobile use, exercise or heavy physical activity, and sleeping with light on had no significant impact on the frequency of sleep problems (p>0.05).

### Predictors of bedtime problems

As shown in Table 4, BMI and age were further analyzed in the regression model to test for the significant predictors of bedtime problems, and both were found to be significant predictors. Older ages (3-, 4- and 5- years) were found to be less likely to have bedtime problems compared with younger age (2- years), whereas underweight was found to be less likely to have bedtime problems compared with normal weight.

**Table 4:** Significant predictors of bedtime problems.

Dependent variable	Bedtime problems	
**Independent variables**	***p*- value**	**OR (95%CI)**
**Age groups**		
3- (years)	0.029	0.449 (0.219 - 0.922)
4- (years)	0.001	0.296 (0.145 - 0.603)
5- (years)	0.034	0.385 (0.159 - 0.929)
**BMI**		
Underweight	0.014	0.373 (0.170 - 0.820)

Each age group was compared with age 2- years, and each BMI group was compared with normal weight group.

### Predictors of wake time on weekdays

Caffeine intake, TV watching, and sleeping with the light on were further analyzed in the regression analysis to test for the significant predictors of wake time on weekdays. Table 5 shows that sleeping with the light on was the only independent factor associated with wake time on weekdays (regression coefficient = −0.847; 95% confidence interval (CI): −1.647 – −0.047; p=0.038).

**Table 5:** Significant predictors of wake time on weekdays.

Dependent variable	Wake Time on Weekdays		
**Independent variables**	**Regression coefficient (β)**	***p*- value**	**95% CI**
Slee ping with light on	−0.847	0.038	(−1.647 - −0.047)

### Predictors of bedtime on weekdays

Caffeine intake, gender, TV watching, and exposure to bright light were further analyzed in the regression analysis to test for the significant predictors of bedtime on weekdays. Table 6 shows that caffeine intake before bedtime was the only independent factor associated with bedtime on weekdays (regression coefficient= 0.559; 95% CI :0.089 - 1.029; p=0.020).

**Table 6:** Significant predictors of bedtime on weekdays.

Dependent variable	Bedtime on weekdays		
**Independent variables**	**Regression coefficient (β)**	***p*- value**	**95% CI**
Caffeine intake	0.559	0.020	(0.089 - 1.029)

### Predictors of bedtime on weekends

Table 7 shows that computer or mobile use before bedtime was associated with later bedtime (regression coefficient=0.711; 95%CI: 0.251-1.171; p=0.003) and that sleeping with light on was associated with earlier bedtime on weekends (regression coefficient=-0.857; 95%CI: -1.612– -0.103; p=0.026).

**Table 7:** Significant predictors of bedtime on weekends.

Dependent variable	Bedtime on weekends		
**Independent variables**	**Regression coefficient (β)**	**p- value**	**95% CI**
Computer or mobile use	0.711	0.003	(0.251 - 1.171)
Sleeping with light on	−0.857	0.026	(−1.612 - −0.103)

### Predictors of regularity of sleep

Logistic regression was performed to test for the predictors of regularity of sleep; it was revealed that age, father’s level of education, mother’s level of education, caffeine intake, and bright light exposure before bedtime were not associated with regularity of sleep on multivariate analysis (p>0.05).

## DISCUSSION

Using the BEARS questionnaire, we conducted this cross-sectional study to investigate sleep disturbances in a sample of Egyptian preschool children attending outpatient clinics. The findings of this study showed that sleep problems were common among this studied sample, as well as poor sleep hygiene. In the present study, more than half of the children had bedtime problems (58.9%), more than one-third of them did not have regular bedtime and wake time (39.5%), one-third of them had frequent awakenings (31%), one-fifth of them had snoring (20.4%), and approximately one-fifth had daytime sleepiness (17.9%).

Our results are consistent with the findings of previous studies reporting a high prevalence of sleep problems among preschoolers.^2,3^ The five most common sleep problems among urban Chinese children under 5 years of age were difficulty falling asleep, nocturnal awakening, bruxism, snoring, and mouth breathing.^2^ The caregivers of 513 Chinese children aged 3-6 years reported that almost 80% (78.8%) of the sample scored above Children’s Sleep Habits Questionnaire (CSHQ) total cutoff, indicating global sleep disturbance. Regarding specific sleep disturbances (CSHQ subscale scores), bedtime resistance (69.5%) and sleep anxiety (56.6%) were the most prevalent , followed by night wakings (34.3%), sleep onset delay (32.8%), and daytime sleepiness (31.1%); parasomnias (19.7%), sleep duration (either too short or too long; 15.1%), and sleep - disordered breathing (14.3%) were comparatively less prevalent among preschoolers.^3^ Another recent study reported that the mean total score of CSHQ was higher in Dutch toddlers aged 2-3 years (indicating more sleep problems) compared with Dutch school-age children.^21^

Van Listenburg et al.^22^ found that bedtime resistance, night awakening and sleep- disordered breathing were more common in the 2–6-year group of Dutch children compared with older age groups, while sleep onset delay, daytime sleepiness, and disorders of sleep duration were more common in older children. Similarly, our study revealed a significant difference in bedtime problems according to age with a higher percentage in 2- and 3- year- old children.

Snoring has been reported in 20.4% of preschoolers, consistent with previous studies reporting a high prevalence of snoring in 1- to 4-year-old children. The prevalence of habitual snoring ranged from 6.6% in 1 years old to 13% in 4 years old.^23^ We found a gender difference in the prevalence of snoring, similar to a previous study that reported habitual snoring in 4.1% of males and 3.3% of females aged 24-71 months.^2^ In our study, there was no statistically significant difference in sleep problems among different BMI groups, except for bedtime problems, as it was the least among underweight (34.3%) compared with those with normal weight, overweight, or obese (62%, 64.8%, 59.7%, respectively). There was no significant correlation between BMI and sleep patterns. These findings agree with those of a previous study that did not find any relationship between sleep duration and good sleeping, which include the number of awakenings and child BMI.^9^

We compared the results of our study to studies that used CSHQ in detecting sleep disorders among preschoolers because of deficiency in studies that used BEARS to detect sleep disturbances among this age group.

In addition, a study conducted to confirm the validity of the Spanish version of BEARS using CSHQ as a comparison instrument revealed that children with sleep problems obtained scores that are significantly higher than children who did not have a sleep problem on the CSHQ-related subscales.^24^ Moreover, several studies that used BEARS revealed its effectiveness in detecting sleep problems among children. ^17,18^

In our study, more than one-third of the children did not have regular bedtime or wake time, and bedtime was at about 10:00 PM. This sleep pattern is not recommended as children of all ages should go to bed before 9:00 PM.^7^ In addition, the sleep schedule of preschoolers showed late bedtime and wake time.

Similarly, Mindell et al.^6^ reported that children and their mothers in the Middle East slept on a shifted schedule, with late bedtimes and wake times compared to those from predominantly Asian and predominantly Caucasian countries/regions.

The factors contributing to inadequate sleep hygiene include factors affecting sleep organization as an irregular sleep-wake schedule; napping too late in the day and an inappropriate sleep schedule; and practices that increase arousal, including lack of bedtime routine, caffeine, and technology use in bedroom.^25^ In the present study, practices that increase arousal was frequently reported among preschoolers as more than two-thirds of the sample watch TV before bedtime, the exposure to bright light and the use of computer or mobile was reported in half of them, caffeine intake and exercise or heavy physical activity was reported in more than one-third of them, in addition to factors affecting sleep organization in the form of irregular sleep schedule.

Our study also demonstrated the negative impact of caffeine intake on sleep as it was associated with frequent awakenings during night with later bedtime. These findings agree with those of Li et al.,^26^ who reported an increase in the number of nocturnal awakenings when children consumed caffeine after 6:00 PM. Similarly, another study found that caffeine consumption was significantly related to sleep routine, morning tiredness, and restless sleep in children, with increasing caffeine consumption correlated with increasing sleep problems.^27^

In agreement with a previous study that showed a negative impact of mobile or computer use at bedtime on the sleep of children,^28^ our study revealed that mobile or computer use near bedtime was associated with later bedtime. Another study reported an association between TV watching near bedtime and more sleep problems in preschoolers.^29^ However, our study showed borderline significant effect of TV watching on bedtime during weekdays.

We found that sleeping with light on was associated with earlier bedtime and wake time among preschoolers and this could be attributed to a decrease in bedtime fear that is common in this age group. Total sleep duration was significantly shorter among preschoolers exposed to bright light before bedtime.

An Egyptian study of sleep patterns and problems among school- age children revealed that paternal illiteracy was associated with higher CSHQ total score and many subscales that may reflect the effect of paternal education on the frequency of sleep disorders.^5^ However in this study, we did not find an association between paternal level of education and sleep problems on multivariate analysis despite the increased regularity of sleep among preschoolers with more educated parents.

The present study had several strengths. First, this study was conducted on tertiary care facility that received patients from different Egyptian urban and rural areas. Second, children’s sleep patterns and problems were reported using simple questionnaire that could be easily used as screening tool in busy clinics. Finally, this study investigated the poor sleep hygiene among this sample, which could be easily corrected by the education of parents about healthy sleep habits as several studies reported the effectiveness of behavioral approaches and sleep hygiene techniques on children’s sleep quality and parents’ quality of life.^30,31^

This study had several limitations. First, the BEARS was not validated, and it was recommended to be compared to accepted gold standards for the diagnosis of pediatric sleep disorders as polysomnography or other pediatric screening tools to assess the validity and sensitivity and specificity of the instrument.^16^ However, it was proved to be an effective simple screening tool in primary care setting in the original pilot study that used it.^16^ Second, children’s sleep was assessed by reports from parents rather than by objective measures, and information bias could not be excluded from this cross-sectional study as there may be parental over- or underestimation of sleep problems.

## CONCLUSIONS

Sleep disorders and poor sleep hygiene are common among this sample of preschoolers attending the pediatric outpatient clinics in a tertiary hospital. This study suggested an association between sleep disorders and age, gender, caffeine intake, bright light exposure, and computer or mobile use before bedtime.
